# Nutritional status and associated factors among the elderly in Guinea: a first national cross-sectional study

**DOI:** 10.1038/s41598-023-42494-3

**Published:** 2023-09-18

**Authors:** Thierno Mamadou Millimono, Alioune Camara, Gustave Mabiama, Mamady Daffé, Farid Boumédiène, Pierre-Marie Preux, Jean-Claude Desport, Philippe Fayemendy, Pierre Jésus

**Affiliations:** 1 Inserm U1094, IRD U270, CHU Limoges, EpiMaCT - Epidemiology of Chronic Diseases in Tropical Zone, Institute of Epidemiology and Tropical Neurology, Univ. Limoges, OmegaHealth, NET - 2 rue du Dr Marcland, 87025 Limoges Cedex, France; 2 Gamal Abdel Nasser University of Conakry, Conakry, Guinea; 3 Department of Family and Home Economics, Advanced Teachers Training College for Technical Education (ATTCTE), University of Douala, Douala, Cameroon; 4 Ministry of Health and Public Hygiene, Food and Nutrition Division, Conakry, Guinea; 5 Resource Centre for Nutrition Nouvelle Aquitaine Region (CERENUT), Isle, France; 6 Nutrition Unit, University Hospital, Limoges, France

**Keywords:** Epidemiology, Ageing, Nutrition

## Abstract

Aging of the Guinean population is a public health concern for the coming years, and the nutritional status of older people is virtually unknown. We also know that this population is growing and that undernutrition and obesity can affect the health of older adults. This study aimed to assess the nutritional status of older people in the general population of Guinea and its associated factors. A representative cross-sectional survey was conducted using sociodemographic, clinical, and anthropometric data (weight and height). Oral status was assessed by using the University of Nebraska Oral Status Scale. Visual acuity was assessed using the Monoyer scale. The standardized prevalence ratio (SPR) of the nutritional status was calculated for each region. The sample included 1698 subjects with a mean BMI of 22.6 ± 4.3 kg/m^2^. A total of 50.3% had impaired oral status and 20.3% had moderately to severely impaired visual acuity. The prevalence of undernutrition was 14.4% and of obesity 5.7%. Differences in the prevalence of nutritional status were found between regions, with an SPR > 1 for undernutrition in the Labé region (SPR 1.9, 95% CI = 1.5–2.5) and for obesity in the Conakry and Kindia regions (SPR of 2.90, 95% CI = 2.0–4.05 and 2.32, 95% CI = 1.5–3.3, respectively). In Guinea, The prevalence of nutritional disorders was approximately 20%. Screening and management of the health and nutritional status of older adults should be a national priority, and management should be adapted to each region of the country.

## Introduction

The number of people aged 60 and over will increase from 1 billion in 2020 to 2.1 billion in 2050, or from 12 to 22% of the world's total population. Every country in the world is expected to experience an increase in the number and proportion of older people^1^. The absolute number of older people in Africa is expected to quadruple between 2010 and 2050, from 56 to 215 million^2^. Little is known about the condition of the elderly in Guinea. The last census in 2014 counted 603,706 people aged 60, and this number is expected to increase to approximately 2 million (6% of the general population) by 2050^3^.

Age is associated with progressive decline in physical function. Appropriate nutrition appears to be one of the key features of successful ageing^4^. Aging is generally associated with an increase in the prevalence of comorbidities and deterioration of nutritional status (undernutrition/obesity)^5,6^.

Undernutrition and obesity in older people are associated with many factors, including inadequate food intake, low levels of education, lack of access to healthcare, tooth loss, hypertension, depression, cancer, and cardiovascular disease^7,8^.

Undernutrition in older adults is associated with low education, older age, physical inactivity, low income, isolation, rural residence, male sex, increased risk of frailty, falls, dependence on activities of daily living, hospitalization, prolonged hospitalization, increased healthcare costs, poor quality of life and high mortality^9,10^. Obesity is associated with functional decline, metabolic syndrome, cardiovascular disease, arthritis, lung abnormalities, urinary incontinence, visual impairment and several cancers^11^. Previous studies conducted in Africa among homebound older people found prevalence of malnutrition ranging from 13.1% to 36.1%^12^.

In Africa, older adults are not prioritized in nutrition intervention programs. Few dieticians and physicians specializing in nutrition work with older people in African countries, and fewer medical professionals conduct research in this area^13^.

In Guinea, the country is currently facing problems related to aging, not because of the low demographic weight of the elderly (about 6% of the total population) but because of their increasing number^14^. Only one study in 2011 focused on assessing malnutrition, but the authors did not define malnutrition; in particular, they did not consider overweight and obesity as malnutrition status^15^. They found a very low prevalence, with 6% of men and 5% of women aged > 65 years being undernourished. In addition, the data were collected from a hospital discharge register and, therefore, could not be extrapolated to the entire elderly population. Finally, they did not consider factors associated with nutritional status.

This study aimed to assess the nutritional status of the elderly population and associated factors in the general population of Guinea.

## Materials and methods

### Modalities of collection

A national observational cross-sectional study of the general population was conducted among community-dwelling, non-hospitalized individuals aged 60 years and older. Data were collected from February to April 2021 in eight administrative regions: the Conakry Governorate, Boké, Kindia, Mamou, Faranah, Kankan, Labé, and Nzérékoré of Guinea. The investigators included 16 medical students, two per region, who were trained in data collection procedures. Participants were informed of the rationale and techniques and signed informed consent forms before proceeding with the study. This study was conducted in accordance with the principles of the Declaration of Helsinki.

The questionnaire was anonymous and pre-tested, and the model questionnaire used was inspired by the study by Torres et al.^16^ in France. The same questionnaire was adapted and used in a general population study conducted in Cameroon 2021^10^. The data were collected from both rural and urban areas.

### Design, sample size and sampling

The prevalence of undernutrition in older people is estimated to be 5.5% in Region^15^. Using a 5% alpha risk, the minimum sample size was estimated to be 1680. Random sampling was used to select elderly subjects. The survey was based on an exhaustive list of enumeration areas obtained from the National Institute of Statistics of Guinea, in which one prefecture/commune was selected, and 210 subjects were selected in each prefecture/commune, that is, 30 enumeration areas per prefecture/commune, with seven subjects in each enumeration area.

A three-stage probability sampling technique was used: first stage (identified prefectures), second stage (enumeration areas) and final stage (people aged 60 and over). In each enumeration areas, the local chief or his representative, who knew the households where the elderly lived, accompanied interviewers. The interviewers went door-to-door until the desired number of people in the enumeration areas was reached.

The main inclusion criteria were to have lived in one of the selected geographical locations for at least six months, to freely agree to participate, and to be able to answer the questions independently or with the help of a family member. Participants were excluded if they did not want to participate, if their family or friends did not want them to participate, or if they were hard of hearing or deaf.

In addition, respondents' age was determined using official documents such as birth certificates, national identity cards, voter cards, marriage certificates, and/or passports, where available and reliable. Age was estimated from the important local events described by the respondent^17^.

### Data collection

The questionnaire was designed and entered into the KoboCollect application (KoBoToolbox, Cambridge, USA). It included sociodemographic data (age, sex, living area, level of education, marital status, main occupation, household size, household size, and source of income) and monthly household income (low, medium, and high) classified according to World Bank indicators^18^. Disease status (cancer, diabetes, cardiovascular disease, tuberculosis, asthma, epilepsy, and HIV infection) and number of medications taken were obtained from statements, and we checked health records to verify the information provided. Blood pressure was measured using an electronic upper-arm blood pressure monitor (Omron™ Paris, France and M3-HEM 7131-E™ Kyoto Japan). Hypertensive individuals were defined by self-report of ongoing treatment, systolic pressure ≥ 140 mmHg, and/or diastolic pressure ≥ 90 mmHg^19^. Three measurements were taken for each arm, and the mean of the last two measurements was used in the analyses. For anthropometric data, height (cm) was measured using a scale graduated to the nearest 0.1 cm; for elderly subjects with standing difficulties, height was obtained from the knee height measured with a Marsden™ 80 cm baby height rod (Paris, France) using the Chumlea formulas^20^. Weight (kg) was measured using a personal scale (Omron HN-288, Paris, France) in 0.1 kg increments. The World Health Organization (WHO) recommendations were used to categorize the nutritional status of elderly subjects on the basis of body mass index (BMI kg/m2): undernutrition, < 18.5; normal weight, 18.5–24.9; overweight, 25.0–29.9; and obese, > 30.0^21^. The WHO standard body mass index classification was used as the dependent variable owing to its simplicity. Oral status was determined using the University of Nebraska Medical Center Oral Status Scale, which uses eight parameters and scores from one (no impairment) to three (significant impairment). This scale categorizes the results obtained into two groups (normal score < 8/24 and abnormal score > 8/24)^22^. Visual acuity was assessed using the Monoyer scale^23^ and WHO visual impairment classification (no visual impairment > 5/10, mild visual impairment < 5/10 to > 3/10, moderate visual impairment 3/10 to > 1/10, and severe visual impairment ≤ 1/10). Visual acuity was measured with both eyes open at a distance of 5 m from the board^23^. All methods were performed in accordance with the relevant guidelines and regulations.

### Statistical analysis

Data were analyzed using SPSS software version 26 (SPSS, Chicago, IL, USA). Continuous variables were expressed as means and standard deviations (SD), and categorical variables were expressed as numbers and percentages (n/%). Normality was tested using the Shapiro–Wilk test. The ANOVA and Kruskal–Wallis tests were used to compare quantitative variables, and the chi-squared test was used to compare categorical variables.

Multivariate analyses were performed to determine the association between nutritional status and the possible associated factors (sociodemographic and medical variables). The dependent variables (undernutrition, normal/overweight, and obesity) were categorized using multinomial logistic regression analysis^24^. Variables with *p* < 0.25 calculated by univariate analysis were included in the multivariate initial model, and a step-by-step descending procedure was used. The threshold of significance was set at *p* < 0.05.

For undernutrition and obesity, we used the Standardized Prevalence Ratio (SPR), which is the ratio between the number of observed and expected cases in a defined population^25^. An SPR >1 indicates that the prevalence of the geographical unit considered is higher than the national one. Conversely, SPR <1 indicates a lower prevalence. An overprevalence was significant when the 95% confidence interval of the SPR was >1 and <1 for an underprevalence. The SPR and their respective 95% CI were calculated separately for undernutrition and obesity and then for undernutrition and/or obesity^26^.

### Ethical approval

The National Committee for the Ethics of Health Research (CNERS) of Guinea approved the research protocol according to Decision No. 012/CNERS/21. Data were collected according to the guidelines of the approved research protocol. Authorizations from administrative services, local authorities, and elderly subjects (consent of the respondent) were obtained.

## Results

### Socio-demographic and health information

A total of 1698 participants aged 60 years and older were interviewed. The mean age was 71.5 ± 9.3 years with a male-to-female ratio of 1.74. No significant differences in age, sex, or area of residence were found among the eight regions studied. Details of the sociodemographic and health information are presented in Table 1.

**Table 1:** General nutritional and health status of the elderly.

	All (n = 1698) n. (%)/mean ± SD	Undernutrition (n = 245) n.(%)/mean ± SD	Normal/overweight (n = 1357) n. (%)/mean ± SD	Obesity (n = 96) n.(%) /mean ± SD	*p*
Age (years)	71.5 ± 9.3	76.0 ± 10.9	71.0 ± 8.9	67.2 ± 6.8	< 0.0001^c^
60–69	819 (48.2)	79 (9.6)	672 (82.1)	68 (8.3)	< 0.0001^a^
70–79	513 (30.2)	68 (13.3)	426 (83.0)	19 (3.7)	
≥ 80	366 (21.6)	98 (26.8)	259 (70.8)	9 (2.4)	
Gender					< 0.0001^a^
Females	619 (36.5)	71 (11.5)	482 (77.8)	66 (10.7)	
Males	1079 (63.5)	174 (16.1)	875 (81.1)	30 (2.8)	
Area					< 0.0001^a^
Urban	601 (35.4)	47 (7.8)	490 (81.6)	64 (10.6)	
Rural	1097 (64.6)	198 (18.0)	867 (79.1)	32 (2.9)	
Region					< 0.0001^a^
Boké	230 (13.5)	32 (13.9)	187 (81.3)	11(4.8)	
Conakry	206 (12.1)	17 (8.3)	155 (75.2)	34 (16.5)	
Faranah	215 (12.7)	39 (18.1)	171 (79.6)	5 (2.3)	
Kankan	205 (12.0)	34 (16.6)	169 (82.4)	2 (1.0)	
Kindia	212 (12.5)	20 (9.4)	164 (77.4)	28 (13.2)	
Labé	210 (12.4)	58 (27.6)	146 (69.5)	6 (2.9)	
Mamou	210 (12.4)	22 (10.5)	186 (88.5)	2 (1.0)	
Nzerekore	210 (12.4)	23 (11.0)	179 (85.2)	8 (3.8)	
Marital status					0.027^a^
Married	1289 (75.9)	186 (14.4)	1041 (80.8)	62 (4.8)	
Not married	409 (24.1)	59 (14.4)	316 (77.3)	34 (8.3)	
Education					0.020^a^
Enrolled	299 (17.6)	215 (15.4)	1111 (79.4)	73 (5.2)	
Not enrolled	1399 (82.4)	30 (10.0)	246 (82.3)	23 (7.7)	
Main activity					< 0.0001^a^
Farmer	631 (37.2)	97 (15.4)	522 (82.7)	12 (1.9)	
Artisan	54 (3.2)	8 (14.8)	43 (79.6)	3 (5.6)	
Shopkeeper	119 (7.0)	5 (4.2)	105 (88.2)	9 (7.6)	
Housewife	149 (8.8)	12 (8.1)	112 (75.1)	25 (16.8)	
Civil servant	68 (4.0)	4 (5.9)	57 (83.8)	7 (10.3)	
Inactive	583 (34.3)	110 (18.9)	440 (75.4)	33 (5.7)	
Retired	94 (5.5)	9 (9.6)	78 (83.0)	7 (7.4)	
Income stability					< 0.0001^b^
No	221 (13.0)	231 (15.6)	1167 (79.1)	79 (5.3)	
Yes	1477 (87.0)	14 (6.3)	190 (86.0)	17 (7.7)	
Average amount					0.0110^a^
< 40€ (< 403,000 FG)	815 (48.0)	137 (16.8)	635 (77.9)	43 (5.3)	
40–80€ (403,000–825,000 FG)	494 (29.1)	70 (14.2)	399 (80.7)	25 (5.1)	
80–180€ (825,000–1,850,000 FG)	226 (13.3)	26 (11.5)	180 (79.7)	20 (8.8)	
> 180€ (> 1,850,000 FG)	163 (9.6)	12 (7.4)	143 (87.7)	8 (4.9)	
Visual pathology					0.4470^b^
No	1250 (73.6)	174 (13.9)	1002 (80.2)	74 (5.9)	
Yes (Myopia. cataract…)	448 (26.4)	71 (15.8)	355 (79.3)	22 (4.9)	
Distance visual acuity (Monoyer)					< 0.0001^a^
Normal	1012 (59.6)	121 (12.0)	821 (81.1)	70 (6.9)	
Moderate	237 (14.0)	37 (15.6)	194 (81.9)	6 (2.5)	
Mild	231 (13.6)	36 (15.6)	185 (80.1)	10 (4.3)	
Severe	218 (12.8)	51 (23.4)	157 (72.0)	10 (4.6)	
Cancers					1.0000^b^
No	1693 (99.7)	245 (14.5)	1352 (79.8)	96 (5.7)	
Yes	5 (0.3)	0 (0.0)	5 (100.0)	0 (0.0)	
Diabetes					0.0010^b^
No	1595 (93.9)	238 (14.9)	1275 (80.0)	82 (5.1)	
Yes	103 (6.1)	7 (6.8)	82 (79.6)	14 (13.6)	
Sickle cell disease					0.7890^b^
No	1592 (93.8)	232 (14.6)	1269 (79.7)	91 (5.7)	
Yes	106 (6.2)	13 (12.3)	88 (83.0)	5 (4.7)	
HBP					< 0.0001^b^
No	1131 (66.6)	184 (16.3)	904 (79.9)	43 (3.8)	
Yes	567 (33.4)	61 (10.8)	453 (79.9)	53 (9.3)	
CVD					0.0160^b^
No	1523 (89.7)	226 (14.8)	1219 (80.1)	78 (5.1)	
Yes	175 (10.3)	19 (10.9)	138 (78.8)	18 (10.3)	
Tuberculosis					1.0000^b^
No	1685 (99.2)	243 (14.4)	1346 (79.9)	96 (5.7)	
Yes	13 (0.8)	2 (15.4)	11 (84.6)	0 (0.0)	
Asthma					0.0130^b^
No	1673 (99.2)	236 (14.1)	1341 (80.2)	96 (5.7)	
Yes	13 (0.8)	9 (36.0)	16 (64.0)	0 (0.0)	
Epilepsy					1.0000^b^
No	1695 (99.8)	245 (14.5)	1354 (79.9)	96 (5.7)	
Yes	3 (0.2)	0 (0.0)	3 (100.0)	0 (0.0)	
HIV					1.0000^b^
No	1679 (98.9)	243 (14.5)	1341 (79.8)	95 (5.7)	
Yes	19 (1.1)	2 (10.5)	16 (84.2)	1 (5.3)	
Surgery history					0.0450^b^
No	1219 (71.8)	187 (15.3)	956 (78.5)	76 (6.2)	
Yes	479 (28.2)	58 (12.1)	401 (83.7)	20 (4.2)	
Health history					0.1070^b^
No	734 (43.2)	103 (14.0)	599 (81.6)	32 (4.4)	
Yes	964 (56.8)	142 (14.7)	758 (78.7)	64 (6.6)	
Medical treatment					0.0040^b^
No	1215 (71.6)	191 (15.7)	966 (79.5)	58 (4.8)	
Yes	483 (28.4)	54 (11.2)	391 (80.9)	38 (7.9)	
Drug intake					0.0090^b^
No	1165 (68.6)	185 (15.9)	923 (79.2)	57 (4.9)	
Yes	533 (31.4)	60 (11.3)	434 (81.4)	39 (7.3)	
Oral status					< 0.0001^a^
Normal < 8	854 (50.3)	97 (11.4)	691 (80.9)	66 (7.7)	
Abnormal ≥ 8	844 (49.7)	148 (17.5)	666 (78.9)	30 (3.6)	
Systolic pressure (mmHg)	118.8 ± 19.6	115.5 ± 20.0	118.9 ± 19.4	125.8 ± 19.7	< 0.0001^c^
Normal systolic pressure	1431 (84.3)	215 (15.0)	1139 (79.6)	77 (5.4)	0.1690^a^
Systolic hypertension	267 (15.7)	30 (11.2)	218 (81.7)	19 (7.1)	
Diastolic pressure (mmHg)	114.5 ± 18.8	111.3 ± 19.2	114.6 ± 18.6	121.1 ± 18.8	< 0.0001^c^
Normal diastolic pressure	1595 (93.9)	215 (13.5)	1286 (80.6)	94 (5.9)	< 0.0001^a^
Diastolic hypertension	103 (6.1)	30 (29.1)	71 (69.0)	2 (1.9)	
Weight (kg)	60.7 ± 12.5	46.8 ± 6.4	61.3 ± 9.8	87.0 ± 11.6	< 0.0001^c^
Height (cm)	163.7 ± 9.2	166.1 ± 9.9	163.5 ± 9.0	160.1 ± 7.9	< 0.0001^c^
BMI (cm)	22.6 ± 4.3	16.9 ± 1.3	22.8 ± 2.8	33.8 ± 3.5	< 0.0001^c^

HBP, high blood pressure; CVD, cardiovascular disease; HIV: human immunodeficiency virus; FG Guinea Francs; a: Chi2 test, b: Fisher test; c: Kruskal–Wallis test.

Most respondents lived in rural areas (64.6%) and did not attend school (82.4%). Respondents were married in 75.9% of cases, working in agriculture in 37.5% of cases and not working in 34.3% of cases. Nearly half of the elderly participants had a net monthly income of less than €40 (403,000 guinean francs). More than half (56.8%) had a medical history, mainly high blood pressure (33.4%), cardiovascular disease (10.3%), and diabetes (6.1%). A total of 30.2% of the subjects took at least five tablets per day, and half of them (50.3%) had an altered oral condition. Respondents declared a visual pathology in 26.4% of cases, and 12.8% had severe visual impairment.

### Nutritional status

The characteristics related to the nutritional status of the elderly population are presented in Table 1. According to the BMI, the prevalence of undernutrition was 14.5%, and of obesity 5.7%. Nutritional status differed significantly depending on age, sex, area of residence, income stability, and activities (*p* = 0.0001 for each). Undernutrition was found in 27.6% and 18.1% of subjects in the Labé and Faranah regions, respectively.

**Figure 1:**
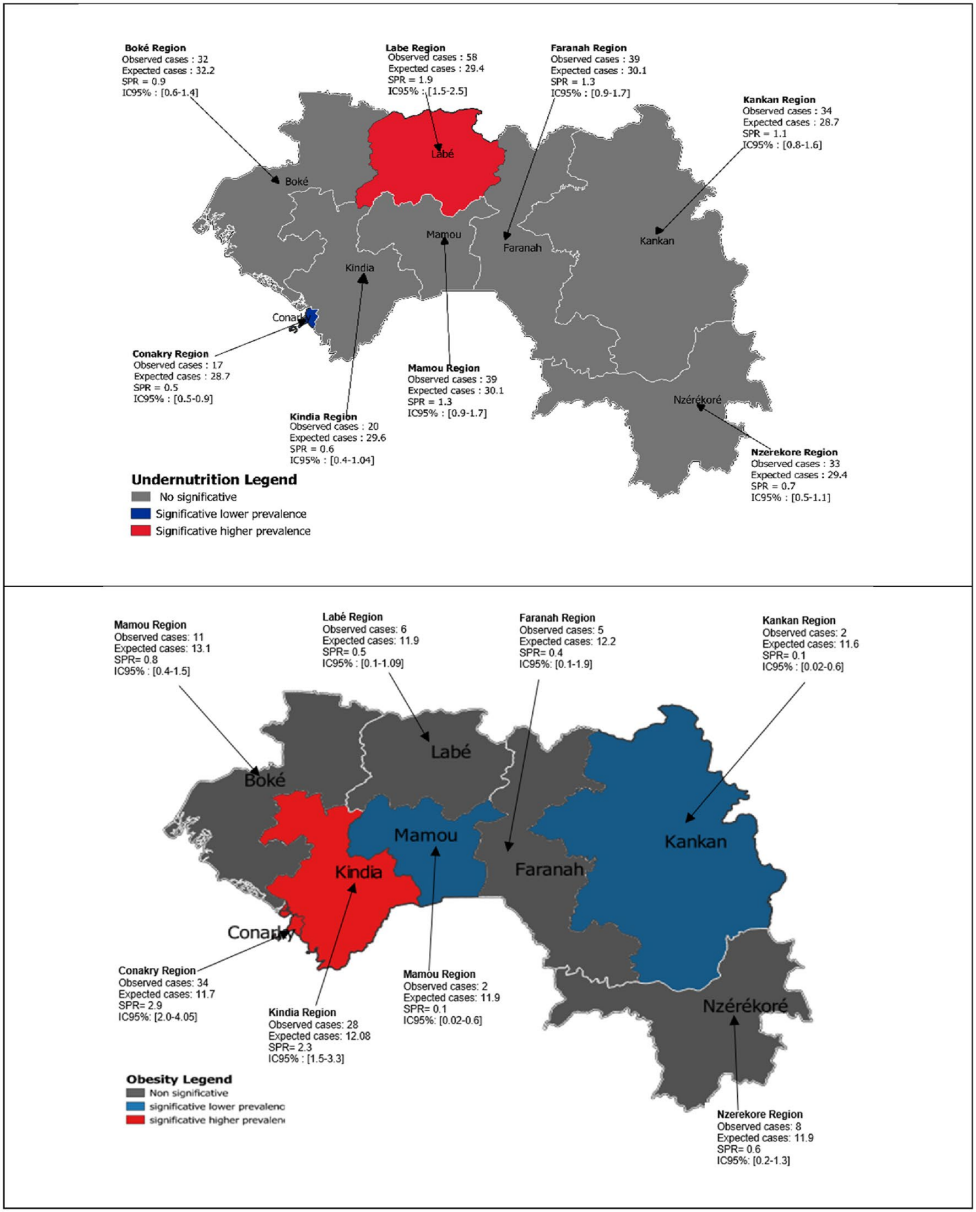
Significant Standardized Prevalence ratios (SPR) of the elderly. The software QGIS v.3.24.0 environment (https://www.qgis.org/) was used to build the Figure.

The SPR showed an overrepresentation of undernutrition in the Labé region (SPR: 1.9, 95% CI = 1.5–2.5, Fig. 1), whereas the Conakry region was underrepresented (SPR: 0.5, 95% CI = 0.5–0.9). For obesity, the Conakry and Kindia regions were overrepresented (SPR: 2.9, 95% CI = 2.0–4.05, and SPR: 2.3, 95% CI = 1.5–3.3, respectively), whereas the Mamou and Kankan regions were underrepresented (SPR: 0.1, 95% CI = 0.02–0.6, respectively).

Univariate analysis of the factors associated with undernutrition and obesity is presented in Table 2. The results of multivariate analysis are presented in Table 3.

**Table 2:** Sociodemographic and health parameters associated with undernutrition and obesity versus normal/ overweight status in univariate analysis.

	Undernutrition vs. normal/overweight			Obesity vs. normal/overweight		
	OR	95% CI	*P*	OR	95% CI	*p*
Age (years)						
60–69	Ref					
70–79	1.3	0.9–1.9	0.083	0.3	0.1–0.6	0.003
≥ 80	3.2	2.3–4.4	< 0.0001	0.4	0.2–0.7	0.002
Gender						
Males	Ref					
Females	0.7	0.5–0.9	0.048	3.9	2.5–6.2	< 0.0001
Area						
Rural	Ref					
Urban	0.4	0.3–0.5	< 0.0001	3.5	2.2–5.4	< 0.0001
Region						
Boke	Ref					
Conakry	0.6	0.3–1.1	0.163	3.7	1.8–7.6	< 0.0001
Faranah	1.3	0.7–2.2	0.271	0.4	0.1–1.4	0.203
Kankan	1.1	0.6–1.9	0.546	0.2	0.04–0.9	0.039
Kindia	0.7	0.3–1.2	0.266	2.9	1.4–6.01	0.004
Labe	2.3	1.4–3.7	0.001	0.6	0.2–1.9	0.490
Mamou	0.6	0.3–1.2	0.212	0.1	.04–0.8	0.028
Nzerekore	0.7	0.4–1.3	0.328	1.9	0.7–0.2	0.564
Marital status						
Not married	Ref					
Married	0.9	0.6–1.3	0.787	0.5	0.3–0.8	0.008
Education						
Not enrolled	Ref					
Enrolled	1.5	1.5–1.5	0.002	0.7	0.4–1.1	0.157
Main activity						
Farmer	Ref					
Artisan	1.0	0.4–2.1	0.998	3.0	0.8–11.1	0.095
Shopkeeper	0.2	0.1–0.6	0.004	3.7	1.5–9.0	0.004
Housewife	0.5	0.3–1.0	0.088	9.7	4.7–19.9	< 0.0001
Civil servant	0.3	0.1–1.0	0.066	5.3	2.0–14.1	0.001
Inactive	1.3	0.9–1.8	0.053	3.2	1.6–6.3	0.001
Retired	0.6	0.3–1.2	0.197	3.9	1.4–10.2	0.006
Income stability						
Yes	Ref					
No	2.6	1.5–4.7	0.001	0.7	0.4–1.3	0.317
Average amount						
< 40€ (< 403,000 FG)	Ref					
40–80€ (403,000–825,000 FG)	0.8	0.5–1.1	0.197	0.9	0.5–2.8	0.765
80–180€ (825,000–1,850,000 FG)	0.6	0.4–1.0	0.081	1.6	0.9–1.5	0.081
> 180€ (> 1,850,000 FG )	0.3	0.2–0.7	0.003	0.8	0.3–1.7	0.630
Visual pathology						
Yes (Myopia. cataract…)	Ref					
No	0.868	0.6–1.1	0.358	1.1	0.7–1.9	0.484
Distance visual acuity (Monoyer)						
Normal	Ref					
Moderate	1.2	0.8–1.9	0.207	0.3	0.1–0.8	0.019
Mild	1.3	0.8–1.9	0.179	0.6	0.3–1.2	0.190
Severe	2.2	1.5–3.1	< 0.0001	0.7	0.3–1.4	0.404
Diabetes						
Yes	Ref					
No	2.1	0.9–4.7	0.050	0.377	0.2–0.6	0.002
Sickle celle disease						
Yes	Ref					
No	1.2	0.6–2.2	0.486	1.2	0.5–3.1	0.622
HBP						
Yes	Ref					
No	1.5	1.1–2.0	0.009	0.407	0.2–0.6	< 0.0001
CVD						
Yes	Ref					
No	1.3	0.8–2.2	0.244	0.4	0.2–0.8	0.010
HIV						
Yes	Ref					
No	1.4	0.3–6.3	0.622	1.1	0.1–8.6	0.904
Surgery history						
Yes	Ref					
No	1.3	0.9–1.8	0.062	1.5	0.9–2.6	0.071
Health history						
Yes	Ref					
No	0.9	0.6–1.2	0.542	0.633	0.4–0.9	0.040
Medical treatment						
Yes	Ref					
No	1.4	1.0–1.9	0.030	0.6	0.4–0.9	0.027
Drug intake						
Yes	Ref					
No	1.4	1.0–1.9	0.020	0.6	0.4–1.0	0.082
Oral status						
Abnormal ≥ 8	Ref					
Normal < 8	0.6	0.4–0.8	0.001	2.1	1.3– 3.3	0.001
Systolic pressure (mmHg)						
Normal systolic pressure	Ref					
Systolic hypertension	1.3	0.9–2.06	0.129	0.7	0.4–1.3	0.341
Diastolic pressure (mmHg)						
Normal diastolic pressure	Ref					
Diastolic hypertension	2.5	1.6–3.9	< 0.0001	0.3	0.09–1.5	0.188

HBP, high blood pressure; CVD, cardiovascular disease; HIV: human immunodeficiency virus; FG Guinea Francs; a: Chi2 test, b: Fisher test; c: Kruskal–Wallis test; Ref : Modality Reference.

**Table 3:** Factors associated with undernutrition and obesity compared to normal status/overweight in multivariate analysis.

	Undernutrition vs. normal/overweight			Obesity vs. normal/overweight		
	OR	95% CI	*P*	OR	95% CI	*P*
Age (years)						
60–69	Ref					
70–79	1.05	0.7–1.5	0.788	0.4	0.2–0.8	**0.008**
≥ 80	1.9	1.2–2.9	**0.002**	0.4	0.1–0.9	**0.033**
Gender						
Males	Ref					
Females	0.6	0.4–0.9	**0.047**	3.6	2.02–6.6	** < 0.0001**
Area						
Rural	Ref					
Urban	0.5	0.3–0.8	**0.006**	1.6	0.8–3.2	0.116
Region						
Boke	Ref					
Conakry	1.6	0.7–3.7	0.248	1.8	0.6–4.9	0.250
Faranah	1.1	0.6–2.07	0.627	0.9	0.2–3.2	0.968
Kankan	0.8	0.4–1.6	0.730	0.5	0.1–2.6	0.446
Kindia	0.8	0.4–1.6	0.577	4.7	1.8–12.1	**0.001**
Labe	3.6	1.9–6.5	< **0.0001**	0.4	0.1–1.5	0.218
Mamou	0.8	0.4–1.6	0.717	0.1	0.03–0.8	**0.030**
Nzerekore	0.7	0.3–1.4	0.423	1.04	0.3–3.4	0.946
Main activity						
Farmer	Ref					
Artisan	1.8	0.8–4.2	0.150	1.1	0.2–4.5	0.853
Shopkeeper	0.4	0.1–1.2	0.147	0.7	0.2–2.2	0.667
Housewife	0.8	0.4–1.7	0.621	2.4	1.01–5.8	**0.047**
Civil servant	0.8	0.2–2.5	0.730	1.7	0.5–5.4	0.368
Inactive	1.3	0.9–2.06	0.105	1.5	0.6–3.4	0.307
Retired	1.08	0.4–2.3	0.835	1.5	0.4–4.6	0.474
Average amount						
< 40€ (< 403,000 FG)	Ref					
40–80€ (403,000–825,000 FG)	1.1	0.7–1.6	0.518	1.2	0.6–2.3	0.482
80–180€ (825,000–1,850,000 FG)	0.9	0.5–1.5	0.775	2.6	1.2–5.5	**0.012**
> 180€ (> 1,850,000 FG )	0.6	0.3–1.3	0.237	1.2	0.4–3.6	0.683
Distance visual acuity (Monoyer)						
Normal	Ref					
Moderate	1.2	0.7–2.07	0.307	0.4	0.1–1.1	0.099
Mild	1.5	0.9–2.4	0.078	1.05	0.4–2.2	0.891
Severe	1.8	1.1–2.9	**0.007**	1.1	0.5–2.5	0.742
Drug intake						
Yes	Ref					
No	1.2	0.9–1.8	0.165	0.5	0.3–0.8	**0.015**
Oral status						
Normal < 8	Ref					
Anormal ≥ 8	1.4	1.0–2.2	**0.047**	0.4	0.2–0.8	**0.021**

HBP, high blood pressure; CVD, cardiovascular disease; HIV: human immunodeficiency virus; FG Guinea Francs; a: Chi2 test, b: Fisher test; c: Kruskal–Wallis test; Ref : Modality Reference.

Significant values are in bold.

In the univariate analyses (Table 2), some factors were associated with undernutrition: age (70–79 years OR=1.3, 95% CI 0.9–1.9, *p *= 0.083 and 80 years OR = 3.2, 95% CI 2.3–4.4, *p* = < 0.0001), female sex (OR = 0. 7, 95% CI 0.5–0.9, *p* = 0.048), urban area (OR = 0.4, 95% CI 0.3–0.5, *p* = < 0.0001), no medication (OR = 1.4, 95% CI 1.0–1.9, *p* = 0.020) and oral status (OR = 0.6, 95% CI 0.4–0.8, *p* = < 0.0001). Factors associated with obesity were age (70–79 years OR = 0.3, 95% CI 0.1–0.6, *p* = 0.003 and 80 years OR = 0.4, 95% CI 0.2–0.7, *p* = 0.002), female sex (OR = 3. 9, 95% CI 2.5–6.2, *p* = < 0.0001), urban area (OR=3.5, 95% CI 2.2–5.4, p= < 0.0001) and married (OR=0.5, 95% CI 0.3–0.8, *p* = 0.008).

Undernutrition (Table 3) was negatively associated with female sex (OR = 0.6 [95% CI 0.4—0.9], *p* = 0.047) and living in an urban area (OR = 0.5 [95% CI: 0.3–0.8], *p* = 0.006). Conversely, living in the Labé region (OR = 3.6 [95% CI 1.9–6.5] *p* =  < 0.0001), being aged 80 years or older (OR = 1.3 [95% CI 1.2–2.9] *p* = 0.002), having severe visual acuity (OR = 1. 8 [95% CI 1.1–2.9] *p* = 0.007) and an abnormal oral status (OR = 1.4 [95% CI 1.0–2.2] *p* = 0.047) were positively associated with undernutrition (Table 3).

Obesity (Table 3) was negatively associated with subjects age 80 years or younger (OR = 0.4 [95% CI 0.1–0.9] *p* = 0.008), subjects aged 70–79 years (OR = 0.4 [95% CI 0.2–0.8], *p* = 0. 008), living in the Mamou region (OR = 0.1 [95% CI 0.03–0.8] *p* = 0.030), not taking any medication (OR = 0.5 [95% CI: 0.3–0.8] *p* = 0.015), and having abnormal oral status (OR = 0.4 [95% CI 0.2–0.8], *p* = 0.021). Conversely, obesity was positively associated with female sex (OR = 3.6 [95% CI 2.02–6.6] *p* < 0.0001) and living in the Kindia region (OR = 4.7 [95% CI 1.8–12.1] *p* = 0. 001), being a housewife (OR = 2.4 [95% CI 1.01–5.8] *p* = 0.047), and having an income of €80 to €180 (825.000 Guinean francs—1.850.000 Guinean francs) (OR = 2.6 [95% CI: 1.2–5.5] *p* = 0.012).

## Discussion

This study aimed to assess the nutritional status of elderly individuals living in communities in Guinea. The majority of the 1,698 elderly subjects were men (63.5%), which is similar to the Guinean national statistics on the elderly population (51%)^14^. Our results differ from those found in the Central African Republic and the Congo, where the majority were females^27^. This could be explained by the variation in the demographic status of the study population and the longer life expectancy of the female population in these countries. However, the distribution of the elderly population according to age group was similar to the national results^14^.

The Rural areas were the most represented (64.6% of persons), which was similar to the results of the Guinean National Survey in 2014 (74%) and in Cameroon (66.4 %)^10,14^.

The prevalence of diabetes (6.1%) and hypertension (33.4%) was lower than that observed in Ghana (diabetes, 11.2%; hypertension, 55.6%)^28^. These diseases can impair visual acuity. In this study, visual pathology in the elderly was 26%, which was lower than that estimated (32%) by the Vision Loss Expert Group of the Global Burden of Disease Study on the acuity of the world's population^29^. This difference in prevalence could be due to genetics, the environment, food insecurity, lifestyle, and access to healthcare.

The higher undernutrition in the Labé region found by multivariate analysis and SPR could be explained by the fact that this region is very far from the capital city of Conakry, with roads often difficult, less agricultural, and an exponential evolution of rural exodus. Elderly individuals living in regions far from the country's capital are known to be the most exposed to the risk of undernutrition^30^.

The higher prevalence of obesity in both the Conakry and Kindia regions, which revealed similar results, was probably due to their almost westernized dietary habits.

A positive association was found between advanced age and malnutrition. A study conducted in Ethiopia indicated that, with advanced age, there is a progressive and generalized loss of skeletal muscle and a reduction in height and bone mass, which could affect the health of elderly subjects^31^. Over the age of 70 years, some weight loss generally occurs, which is attributed to the aging process. This mandatory weight loss is reflected in the higher levels of underweight subjects associated with age^30^.

In our study, severe visual acuity was positively associated with malnutrition. We found no studies on the relationship between nutrition and visual acuity in Africa. Several studies have shown that the prevalence of visual impairment increases significantly with age and that vision is poorer in women than in men of the same age^32^.

Abnormal oral status is positively associated with undernutrition. Denaturation of oral health is a risk factor for lack of appetite in the elderly population, especially the loss of teeth, which limits the ability to grind solid food. A study in Brazil by Renato et al. on the oral health of independent elderly subjects reported that almost half of them had poor oral health and were at a higher risk of malnutrition^33^. Another study in Brazil reported poor oral health in the elderly due to partial or complete loss of teeth^34^. This is an important parameter to be considered in future nutritional interventions.

Female sex was negatively associated with malnutrition. This could be explained by the fact that most elderly women do not work, are housewives, and are assisted by their children, daughters-in-law, and grandchildren.

A positive correlation was observed between obesity and residence in the Kindia region. Obesity was also higher in the Conakry region in SPR, but multivariate analysis did not find any correlation between them. The Kindia region is an active citrus production region near the capital city of Conakry. In these two regions, the phenomenon of nutritional transition is probably implied by changes in eating habits, westernization of the diet, and a sedentary lifestyle^31^. Similarly, Jesus et al. found that living in the capital of Congo, Brazzaville, was positively associated with obesity in this country^27^.

Being a housewife was positively associated with obesity. This study is similar to that of Silviera et al. in Brazil^35^. In addition, housewives in Guinea are usually assisted by foster children, daughters, granddaughters, and even daughters-in-law in performing household chores, resulting in low physical activity and a sedentary lifestyle.

A monthly income of €80–180 was positively associated with obesity. Considering the guaranteed minimum interprofessional wafer in Cameroon of €55 per month, the results suggest that a higher income facilitates the development of obesity^10^. In Africa, urbanization and a higher income may lead to westernization of diet and a decrease in physical activity^36^.

Living in the Mamou region was negatively associated with obesity. The main activities in this region are trade and market gardening, which are also carried out by elderly subjects, possibly preventing them from becoming sedentary and giving them the opportunity for a varied diet.

Obesity is negatively associated with the absence of drugs. Our study corroborates a review by Mabiama et al. for the whole of Africa^37^. Obesity is associated with chronic diseases, such as diabetes, hypertension, and dyslipidemia, which leads to an increase in drug intake^38^. The lack of pill-taking in elderly subjects could be related to the absence of a pathology or the inability to pay for treatment^39^.

We observed a negative correlation between abnormal oral hygiene and obesity. Good oral health helps people to eat better and may prevent obesity^33^; however, in Guinea, elderly subjects often tend to use toothpicks, the cost of oral health care is high, and dentists may be scarce in some areas of the country.

Being aged ≥ 70 years was negatively associated with obesity. This result is similar to that of Jesus et al. in the Republic of Central Africa and Congo. Aging is often accompanied by chronic diseases and a decrease in food intake, which leads to the loss of weight and fat, thus exposing the elderly population to undernutrition and protection against obesity^27,40^.

### Strengths and limitations

The strength of this study is that it is the first representative national study of elderly subjects in all administrative regions with a large number of subjects surveyed at home. This is also the first study to use SPR in nutrition, which discriminates regions in the country with nutritional risk.

We encountered difficulties during the conduct of the study due to the COVID-19 pandemic and Ebola virus outbreak in the administrative region. However, in this cross-sectional study, we were unable to assess COVID-19 and Ebola in the study population.

### Public health implications

The data obtained constitute a reliable basis for knowledge of the health and quality of life of elderly people in Guinea. The study shows that the measurement of BMI and the techniques for evaluating oral status and visual acuity are easy to use and inexpensive tools. They could be implemented, for example, during surveys carried out every five to ten years, with the aim not only of screening and treatment of nutritional disorders, but also of improving the oral condition and visual abilities of elderly people, and therefore their quality of life.

The results of the SPR can help better guide public health decisions, the Labé region being the most affected by undernutrition and the Conakry and Kindia regions the most affected by obesity.

Knowing that the nutritional status of hospitalized elderly people is usually worse than in the general population, and that there is no data on this subject in Guinea, a survey similar to that of the present study could also be carried in hospitals.

## Conclusion

This is the first cross-sectional study on the nutritional status of community-dwelling older adults. In this nationally representative study of the elderly population in Guinea, the prevalence of undernutrition was 14.4%, which was higher than in the 2021 study. Undernutrition was positively associated with urban areas, age ≥ 80 years, severely decreased visual acuity, and abnormal oral status. Obesity was 6.1%, higher in urban areas, and positively correlated with the female sex, being a housewife, and having a large income for this country (€80 to €180).

## Data Availability

The data collected were located at the Institute of Epidemiology and Tropical Neurology, Inserm U1094, IRD U270, University EpiMaCT—Epidemiology of chronic diseases in tropical zones, OmegaHealth, University Limoges, France. Data are available upon reasonable request from the corresponding author.

## Abbreviations

BMI: Body Mass Index
CI: Confidence interval
OR: Odds ratio
SPR: Standardized prevalence ratio
